# Isolating endogenous visuo-spatial attentional effects using the novel visual-evoked spread spectrum analysis (VESPA) technique

**DOI:** 10.1111/j.1460-9568.2007.05968.x

**Published:** 2007-12

**Authors:** Edmund C Lalor, Simon P Kelly, Barak A Pearlmutter, Richard B Reilly, John J Foxe

**Affiliations:** 1 The Cognitive Neurophysiology Laboratory, Nathan S. Kline Institute for Psychiatric Research, Program in Cognitive Neuroscience and Schizophrenia Orangeburg, NY 10962, USA; 2 The Cognitive Neurophysiology Laboratory, St Vincent's Hospital Fairview Dublin, Ireland; 3 School of Mechanical, Electrical and Electronic Engineering, University College Dublin Dublin, Ireland; 4 Program in Cognitive Neuroscience, Department of Psychology, City College of the City University of New York 138th Street & Convent Avenue, New York, NY 10031, USA; 5 The Hamilton Institute, National University of Ireland Maynooth Co. Kildare, Ireland

**Keywords:** EEG, P1, split spotlight, visual evoked potential

## Abstract

In natural visual environments, we use attention to select between relevant and irrelevant stimuli that are presented simultaneously. Our attention to objects in our visual field is largely controlled endogenously, but is also affected exogenously through the influence of novel stimuli and events. The study of endogenous and exogenous attention as separate mechanisms has been possible in behavioral and functional imaging studies, where multiple stimuli can be presented continuously and simultaneously. It has also been possible in electroencephalogram studies using the steady-state visual-evoked potential (SSVEP); however, it has not been possible in conventional event-related potential (ERP) studies, which are hampered by the need to present suddenly onsetting stimuli in isolation. This is unfortunate as the ERP technique allows for the analysis of human physiology with much greater temporal resolution than functional magnetic resonance imaging or the SSVEP. While ERP studies of endogenous attention have been widely reported, these experiments have a serious limitation in that the suddenly onsetting stimuli, used to elicit the ERP, inevitably have an exogenous, attention-grabbing effect. Recently we have shown that it is possible to derive separate event-related responses to concurrent, continuously presented stimuli using the VESPA (visual-evoked spread spectrum analysis) technique. In this study we employed an experimental paradigm based on this method, in which two pairs of diagonally opposite, non-contiguous disc-segment stimuli were presented, one pair to be ignored and the other to be attended. VESPA responses derived for each pair showed a strong modulation at 90–100 ms (during the visual P1 component), demonstrating the utility of the method for isolating endogenous visuo-spatial attention effects.

## Introduction

Much of what is currently known about the mechanisms and effects of visual spatial attention in the brain is owed to advances in modern electrophysiological and hemodynamic imaging techniques (e.g. Kanwisher & Wojciulik, 2000; Luck *et al.*, 2000). Of these techniques, the event-related potential (ERP) method has been most informative in uncovering the temporal dynamics of attentional processes in humans. For example, the longstanding issue of whether spatial selection can occur at the early sensory level, and not only at later levels of decision-making and response programming, has seen major steps toward resolution using ERPs (e.g. Luck *et al.*, 1994; Hillyard & Anllo-Vento, 1998). Typically in such studies a comparison is made between the responses to a stimulus when attended vs when unattended. Because the responses to each of multiple stimuli presented together cannot be separated in standard ERP data, stimuli must be presented in isolation to the attended or unattended space at different times. This represents a serious limitation of the technique, in that it is not possible to emulate the more common real-life situation where both relevant and irrelevant information are present in the visual field at the same time.

Aside from having lower ecological validity, there is a fundamental limitation that stems from the stimulus presentation constraints in ERP attention paradigms. This issue arises in designs attempting to assess the physiological effects of endogenous attention using paradigms in which suddenly onsetting stimuli appear in either a cued (and hence attended) or uncued (unattended) location in space. Specifically, the sudden onset of the stimuli may actually ‘grab’ attention exogenously, either assisting the subject in attending to the cued location or causing involuntary, transient reorienting from cued to uncued or ‘distracter’ locations, a phenomenon for which there exists a long line of evidence (e.g. Jonides, 1981). Thus, it is not certain that the observed modulations of electrophysiological responses to ‘endogenously’ cued stimuli solely reflect endogenous attentional influences.

Several ERP studies have attempted to disentangle the interactions between endogenous and exogenous attention on visual processing through manipulation of various aspects of the experiment design. For example, the use of centrally and peripherally located cues to emphasize endogenous and exogenous attention, respectively, has been reported (Fu *et al.*, 2005; Hopfinger & West, 2006). Manipulation of the likelihood of stimuli appearing at particular locations has also been used to emphasize endogenous vs exogenous attention (Natale *et al.*, 2006). However, all of these experiments utilize suddenly onsetting stimuli to measure endogenous and exogenous attention alike and, thus, are still somewhat confounded.

The VESPA (visual-evoked spread spectrum analysis) technique recently developed by our group (Lalor *et al.*, 2006) allows for the measurement of electrophysiological activity in response to each of several simultaneously presented stimuli with high temporal resolution, thus overcoming the abovementioned limitations. In this paper we aim to utilize this VESPA method to establish the time range over which electrophysiological effects of sustained endogenous attention to a visual stimulus are seen while ignoring another concurrent visual stimulus.

## Materials and methods

### Subjects

Fifteen healthy subjects (three female) aged between 20 and 41 years participated in the study. All had normal or corrected-to-normal vision. The experiment was undertaken in accordance with the Declaration of Helsinki, and the Ethics Committee of St Vincent's Hospital in Dublin, Ireland approved the experimental procedures and each subject provided written informed consent.

### Experimental paradigm

**Fig. 1:**
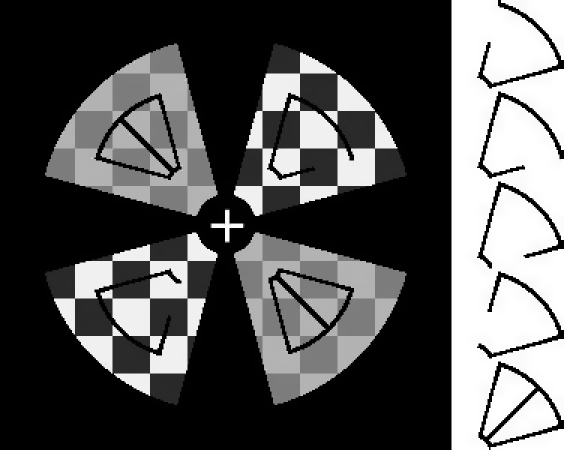
(left panel) Stimulus setup showing UL–LR stimulus at one contrast level and LL–UR stimulus at another contrast level. Target symbols are shown in the UL–LR stimulus. (right panel) The symbols displayed in the upper right segment, with the target symbol for all runs shown at the bottom.

The stimulus configuration is shown in Fig. 1 (left panel). It consisted of four checkerboard-patterned segments of an annulus, the inner diameter of which subtended 1° of visual angle and the outer diameter 7°. The refresh rate of the monitor was set to 60 Hz and, during trials, the contrast of the checkerboard pattern in each segment was modulated on every refresh by a stochastic waveform to facilitate the estimation of the VESPA (Lalor *et al.*, 2006; see also VESPA analysis section below). For all of the trials the upper-left and lower-right segments (‘UL–LR’) were modulated together using one signal, and the lower-left and upper-right segments (‘LL–UR’) using another. Thus, the four segments can be considered as two pairs.

A sequence of black symbols from a set of five, one of which was defined as the target (Fig. 1 right panel), was superimposed on the center of each checkerboard segment. This target could be identified by the presence of a diagonal bar running through its center. Subjects were required to respond with a button press on detection of target symbols appearing in both attended segments simultaneously (‘valid’), while ignoring the opposite pair of segments (‘invalid’). A different stream of symbols was displayed in each of the four segments at all times, with a change in the symbols occurring every 11 frames, i.e. every 183 ms, with no interval between symbols. It has been reported that the minimum time needed to identify a target at one location and then switch attention to identify a target at a second location is in the range of 200–500 ms (Müller *et al.*, 1998). The brief durations used in the present study suggest therefore that it would be impossible to achieve a high rate of target-pair detections using a switching strategy and that accurate performance could only be achieved by dividing attention between the two locations. Target symbols occurred equally often in all segments. If a target symbol appeared in only one segment of a pair, it was guaranteed that a target symbol would not appear in the other segment of that pair on the next change. Target-pairs appeared equally often at random intervals in both attended and unattended segment-pairs and were separated in each pair by a minimum of 733 ms, with a mean (± SD) rate of 8.5 (± 2.5) target-pairs per segment-pair per trial (i.e. one every 4.7 s on average). A video illustrating the experiment can be seen in the Supplementary material (Video S1).

Each subject underwent a total of 20 runs, each consisting of five trials. Each trial lasted 40 s after which there was a 20 s break before the next trial. During the break periods the setup shown in Fig. 1 left panel was displayed on the screen with the corresponding targets displayed in each segment and no modulation of the contrast of the checkerboard segments. Prior to the start of every trial (1200 ms) a warning stimulus was presented, wherein the targets in one pair of segments flickered off for 200 ms, on for 800 ms and back off for the remaining 200 ms indicating the segment-pair to be attended during the upcoming trial. The pair of segments to be attended to alternated with every trial with the initial pair counterbalanced across subjects.

### Behavioral performance

Because the paradigm did not involve a simple ‘go/no-go’ task, the sequence of stimuli had to be examined carefully in order to determine whether or not a button press was actually made in response to the presentation of a double-target in the to-be-attended segments. For example, while a button press may have occurred after the presentation of a double-target in the to-be-attended segments, it is possible that it was actually in response to a double-target in the to-be-ignored segments and that the subject was in fact not complying with the task. To fully account for this possibility, the data were examined in two ways.

In the first analysis all responses occurring in the interval 200–1000 ms following target symbols appearing in any segment were accounted for in the following order:

1. valid double-target
2. valid single-target
3. invalid double-target
4. invalid single-target

Responses counted towards each successive category were excluded from the remaining categories. In the second analysis, the order was rearranged so that the invalid targets were considered first.

### Eye movement control

Subjects were required to maintain central fixation during all trials. While we reasoned that the nature of the divided attention task would be such as to discourage eye movement given the rapid rate of change of the symbols (183 ms), electrooculographic (EOG) data were recorded and examined to explicitly monitor this. Horizontal EOG (HEOG) was recorded from the outer canthi of the eyes and each was referenced to the nasion. During a trial, if the HEOG for one eye was above 16 µV concurrent with the HEOG for the other eye being below −16 µV a deviation from fixation was deemed to have occurred and the trial was rejected. This limit, which may be considered conservative in light of the experimental design, resulted in a mean rejection rate of 15% of trials.

### Electroencephalographic (EEG) acquisition

EEG data were recorded from 72 electrode positions referenced to location Fz, filtered over the range 0–134 Hz and digitized at a rate of 512 Hz using the BioSemi Active Two system. Offline, the EEG was digitally filtered with a passband of 1–35 Hz.

### VESPA analysis

The VESPA is an estimate of the first order impulse response of the visual system (Lalor *et al.*, 2006). It is based on the assumption that the EEG response to a stimulus whose luminance or contrast is rapidly modulated by a stochastic signal consists of a convolution of that signal with an unknown impulse response. That is, we assume that the known rapid stimulus modulation signal causes correlated activity in the EEG. Thus, a reverse correlation procedure facilitates estimation of the impulse response, which, when convolved with the input stimulus signal, approximates the output EEG. This impulse response function is known as the VESPA. As mentioned above, in the present study the contrast of the checkerboard patterns in each segment was modulated on every refresh of a monitor set to 60 Hz. The signals that controlled these contrast modulations had their power spread uniformly over the range 0–30 Hz. It is important to realize that the VESPA is not a response to the onset of any particular discrete event. It is derived over a period of time from the continuous stimulus and the concurrently recorded EEG. The VESPA itself is plotted on a time axis that indicates the relationship between the incoming stimulus signal at any particular time and the output EEG a certain fixed time later. It should also be noted therefore that the estimation of the VESPA in this study is unrelated to the symbols superimposed on the modulating checkerboard segments.

The profiles of these VESPAs are highly correlated with those of transient visual evoked potentials (VEPs). However, unlike the transient VEP, multiple stimuli driven by orthogonal modulating signals can be simultaneously presented within a visual display and a separate VESPA can be derived for each. It is not necessary for these signals to have differing frequency distributions, they can simply be different instantiations of the same random process, i.e. they can have exactly the same statistics. In the present study, using the data from every 40-s trial and the corresponding contrast modulation signals, we simultaneously derived a VESPA for each of two pairs of non-contiguous disc segments, while subjects attended to one pair and ignored the other. We then averaged VESPA responses for each pair and condition across trials. The same two stochastic signals were used for all trials. The assignment of signals to segment-pairs was switched after 10 runs, with the starting assignment counterbalanced across subjects.

VESPAs were measured using a sliding window of 500 ms of data starting 100 ms prestimulus. This results in a VESPA plotted on a time axis from −100 ms to 400 ms. Therefore, the VESPA at −100 ms, for example, indexes the relationship between the input noise signal and the EEG 100 ms earlier; obviously this should be zero. As another example, the VESPA at 100 ms indexes how the input noise signal affects the EEG 100 ms later.

In an effort to improve the signal-to-noise ratio (SNR) of the VESPAs we carried out blind source separation of the data as a pre-processing step. This resulted in an improvement in SNR for the average VESPA, from 12.9 dB to 17.7 dB, where the SNR was calculated by defining the noise as the mean of the squared values in the 100-ms interval immediately preceding the stimulus, and the signal as the mean of the squared values in the interval 35–175 ms post-stimulus. The details of the blind source separation method are provided in the supplementary Appendix S1.

## Results

### Behavioral performance

The behavioral performance on the task, averaged across all trials and subjects is shown in Table 1 and confirms that subjects were deploying their attention correctly. The first two columns list the percentage of 200–1000-ms intervals following the presentation of targets that contained a response, when the to-be-attended segments were considered first, i.e. the first analysis outlined above. In this case, when the interval following the presentation of a target-pair in the to-be-attended segments was checked for a response, one was present in 68% of cases. In contrast, when the to-be-ignored segments were considered first (third and fourth columns), just 10% of intervals following a target-pair in the to-be-ignored segments contained a response. There was no significant difference in behavioral performance between attending to the UL–LR segments and the LL–UR segments *F*_1,14_ = 0.38, *P* > 0.5.

**Table 1:** Behavioral results averaged across subjects

	To-be-attended first		To-be-ignored first	
		
	UL–LR	LL–UR	UL–LR	LL–UR
To-be-attended double	68%	67%	15%	15%
To-be-attended single	3%	3%	10%	9%
To-be-ignored double	∼0%	∼0%	10%	10%
To-be-ignored single	∼0%	∼0%	∼0%	∼0%

The first two columns list the performance determined by accounting for responses to valid segments first, and the second two columns list that determined by accounting for responses to invalid segments first.

### Attentional modulation of the VESPA

**Fig. 2:**
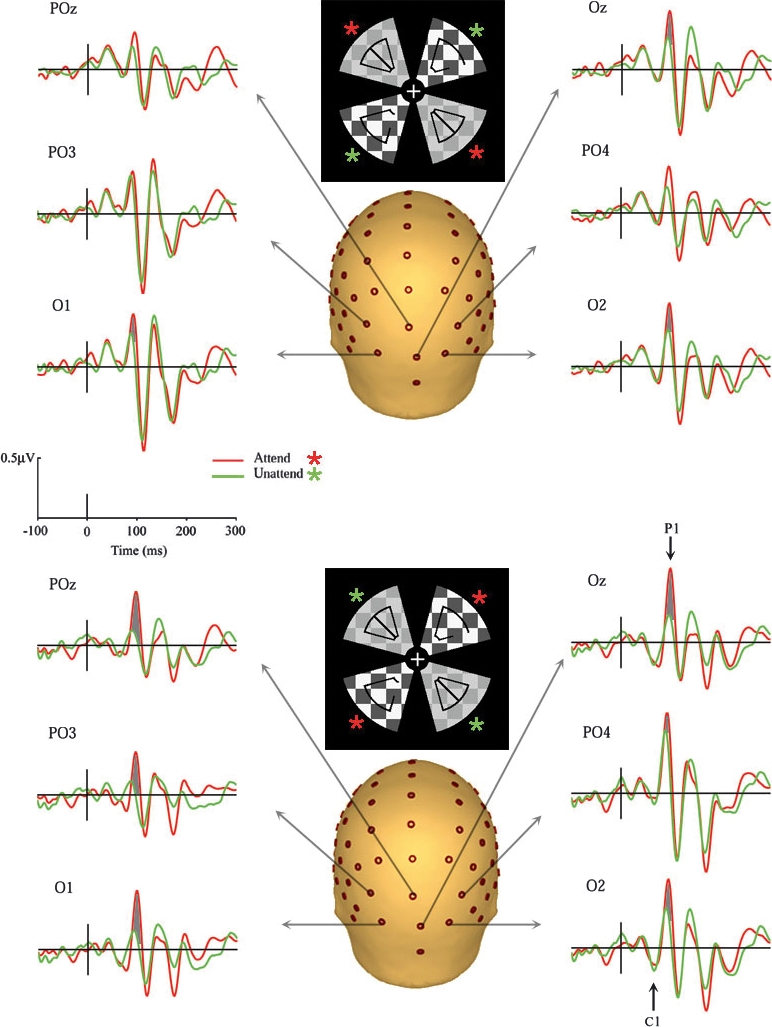
Plots of the VESPAs to the attended and unattended stimuli for six occipito-parietal electrodes for both the UL–LR (upper panel) and LL–UR (lower panel) trials. Significant P1 attention effects are highlighted in gray.

**Fig. 3:**
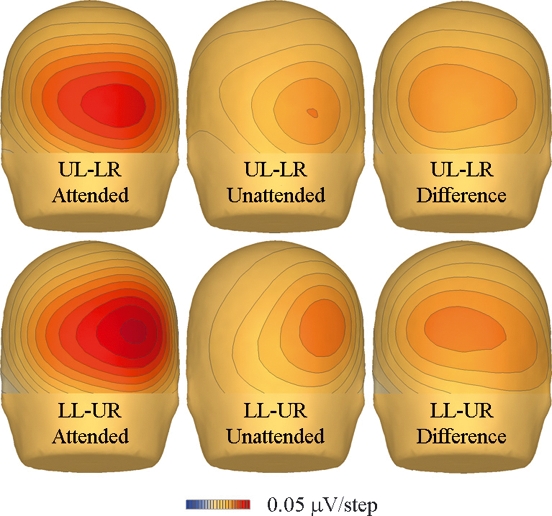
Scalp topographies of the attention effects averaged across the 90–100 ms range (P1), and across all subjects for both the UL–LR (upper panel) and LL–UR (lower panel) stimuli for the attended and unattended cases, and the difference between the attended and unattended cases.

Figure 2 shows the group average VESPAs for the UL–LR and LL–UR stimuli, in the cases where they were attended and unattended, for six representative parieto-occipital electrode locations. Because of the prevalence of attentional modulations of the P1 component in the VEP literature, we first wished to test the hypothesis that a similar effect would be seen with the VESPA. We defined the P1-dependent measure as the average amplitude in the interval 90–100 ms, selected on the basis of peak latencies in group-average waveforms. Attentional modulations of the P1 component were statistically tested first via an omnibus 2 × 2 × 3 × 3 anova with factors of stimulus (UL–LR vs LL–UR), attention (UL–LR vs LL–UR), region (three levels; left: O1/PO3/PO7; midline: Oz/POz/Pz; and right: O2/PO4/PO8 scalp) and electrode (three levels; O1/Oz/O2; PO3/POz/PO4; and PO7/Pz/PO8). We restricted our analysis to these electrode locations based on the scalp distribution of VESPA amplitude being confined to posterior regions (Fig. 3).

Confirming the principal hypothesis of the study, a strong interaction was found between stimulus and attention (*F*_1,14_ = 9.32, *P* < 0.01). As can be seen in Fig. 2, this was driven by greater VESPA P1 amplitude when each stimulus was attended compared with when unattended. A main effect of electrode (*F*_2,28_ = 8.96, *P* < 0.005) and a marginal effect of region (*F*_2,28_ = 3.89, *P* = 0.055) alluded to the topographic specificity of the P1.

Also, a four-way interaction between all factors (*F*_4,56_ = 3.94, *P* < 0.01) indicated a degree of topographic specificity in the attentional modulation of the P1. *Post hoc* comparisons revealed that the effect of attention on LL–UR stimuli reached significance at all electrodes across the three regions (*P* < 0.05), and on UL–LR stimuli at electrodes O1, PO7, Oz, O2 and PO8 (all *P* < 0.05; highlighted in gray in Fig. 2). To test for a possible hemispheric bias of the P1 attentional modulation, a follow-up anova was carried out using the attended-minus-unattended difference as the dependent measure, with the two factors of stimulus (UL–LR vs LL–UR) and hemisphere (left: PO3; right: PO4). However, no interaction was found (*F*_1,14_ = 0.06, *P* > 0.5).

The ‘N1’ and ‘P2’ components, measured as the average amplitude in the interval 105–120 ms and 130–145 ms, respectively (see Fig. 2), were subjected to the same omnibus anova as the P1. In both cases, no interaction was found between stimulus and attention (‘N1’: *F*_1,14_ = 0.19, *P* > 0.5; ‘P2’: *F*_1,14_ = 0.18, *P* > 0.5).

## Discussion

In this study we have quantified, with detailed temporal precision, the electrophysiological effects of endogenous attention to a subset of non-contiguous, simultaneous, continuously presented stimuli. Our paradigm avoids using suddenly onsetting stimuli and, in so doing, avoids bringing confounding exogenous attentional effects into play. We found a strong attentional enhancement of the amplitude of the VESPA in the range of the P1 component (90–100 ms), indicating that endogenous attention occurs during the early stages of sensory processing.

Modulation of the P1 component of the transient VEP in visual spatial attention studies has been reported widely (e.g. Hillyard *et al.*, 1995; Müller & Hillyard, 2000). Unlike other studies in which the amplitude of multiple N1 (140–200 ms) and/or later components (N2, P300) of the transient VEP have been enhanced with attention (Mangun & Hillyard, 1987; Rugg *et al.*, 1987; Heinze *et al.*, 1990), no enhancement of VESPA components other than the P1 was observed here. While the waveforms for the LL–UR stimulus in Fig. 2 suggest that there may be attentional enhancements of the negative peak at 175 ms, this did not appear to be the case for the UL–LR segments, and no significant effects were found.

The notion that only endogenous, and not exogenous, attentional orienting mechanisms are called upon in this paradigm may be the reason for the restricted timeframe of the modulations found in the present study. Our finding is consistent with Natale *et al.* (2006), who reported that attentional modulation of the P1 component of the VEP reflected endogenous mechanisms, while modulation of the N1 component represented exogenous attention. They suggest that the P1 effect might index an attentional facilitation of early sensory processing while the N1 effect may index exogenous orienting of attention, possibly representing activity of frontal and parietal components involved in eliciting attention changes. This finding contrasts with the study of Hopfinger & West (2006) who reported that exogenous attention dominated the late phase of the P1 component, regardless of where endogenous attention had been orientated and that endogenous attention dominated a later, higher-order stage of processing indexed by an enhancement of the P300 that was unaffected by exogenous attention. These conflicting findings further highlight the difficulty in disentangling endogenous and exogenous attention effects using suddenly onsetting stimuli. It is worth noting that a direct comparison of VESPA and standard transient VEP componentry may not be straightforward; one apparent difference is that the succession of components of alternating polarity appears to proceed in a shorter timeframe in the VESPA. However, as we have not measured transient VEPs from corresponding stimuli here, this cannot be judged definitively. In fact, differing behavior between the ‘P1’ components in the VEP and VESPA has already been seen in a recent study on schizophrenia, suggesting that in each case the P1 reflects activity of a distinct subpopulation of cells (Lalor *et al.*, 2007). Future work will aim to elucidate the relationship between VESPA components and those of the transient VEP. For example, examining the retinotopic sensitivity of components of the VESPA (e.g. polarity inversion of the earliest C1-like component for upper- vs lower-field stimuli) will help to establish their cortical origins, as has been done with the standard VEP (Di Russo *et al.*, 2002).

Another possible explanation for the narrow timeframe of the attention effect in the present study is that the task demands inherent in the paradigm may be such that selection is required only at this level. The timeframe of the P1 (90–100 ms) may be the critical point at which targets can be fully classified on the basis of the presence of centrally located diagonal bars within the symbols in both non-contiguous locations. That selection could be so finely titrated in the system may also be particular to the situation where stimuli are continuously and/or simultaneously presented.

The scalp distributions of Fig. 3 provide further illustration of the attentional modulations during the P1 timeframe. These modulations were restricted to a relatively small number of posterior electrode sites. Somewhat surprisingly, the VESPA P1, which has previously been shown to be restricted to a unimodal midline occipital focus, displays a similar unimodal focus in this study, despite the paired stimuli. It is also interesting to note that the distribution of the difference between the attended and unattended cases, i.e. the attentional effect, is quite different from that of the response to the stimulus itself. Though this appears at odds with the common finding of matched topographies in the difference (attended minus unattended) and unattended conditions (Hillyard & Anllo-Vento, 1998), it must be considered that the modulations here are measured from continuous stimuli and thus may receive contributions from control processes as well as the expression of sensory biasing. This idea will be investigated in further work.

The ability to assess the timing of electrophysiological effects of attention using displays containing multiple, continuously presented elements has other advantages. It allows for the design of more ecologically valid experimental paradigms and the investigation of questions that were hitherto very difficult to answer conclusively, and it also addresses the practical issue that certain attention experiments carried out using functional magnetic resonance imaging (fMRI) have not been replicable in ERP designs. In fMRI experiments responses to each of multiple stimuli can be isolated in retinotopic visual areas with high spatial resolution (e.g. McMains & Somers, 2004). However, the low temporal resolution of fMRI precludes examination of the precise temporal dynamics of the attentional effects. The activation of the lower-tier visual areas suggests that attention may be expressed in a modulation of activity at the earliest hierarchical stages; however, it is possible that the modulation of activity in those areas reflects feedback of later processing (e.g. Martínez *et al.*, 1999). One example is the study of McMains & Somers (2004) on the splitting of the attentional spotlight. This study employed multiple simultaneously presented stimuli to show that attention divided over non-contiguous regions of space is expressed in low-level visual areas. Similar split-spotlight effects were seen using the steady-state visual-evoked potential (SSVEP) technique (Müller *et al.*, 2003), which, like fMRI, lacks the ability to resolve the temporal profile of modulation.

The results of the present study address this issue of the timing of the division of visual spatial attention. The experimental paradigm based on the VESPA has allowed us to analyse, in detail, the timing of attentional effects on responses to stimuli in non-contiguous regions of space. It should be noted that in our stimulus configuration there was no intervening distracter between the regions to be attended. As a result, there is the possibility that subjects, rather than splitting their attention, may have performed the task by elongating a unitary spotlight into such a formation as to encapsulate the regions where the targets were expected to appear, while remaining spatially contiguous through the fixation point. However, in a follow-up to their earlier study, McMains & Somers (2005) investigated the processing efficiency of split-spotlight attention mechanisms compared with that of a ‘zoom lens’ unitary beam. Using behavioral measures and retinotopic BOLD signal activation amplitude, the authors concluded that, when attention is spread over the same spatial extent, there is significant benefit for dividing attention. Given the difficulty of the task described in the current study and the successful performance of that task by the subjects, it is likely that the subjects sought to harness this benefit by splitting their attentional spotlight. Müller *et al.* (2003) also demonstrated improved behavioral performance for the split-spotlight case compared with attention to adjacent stimuli, which again points to spotlight division as an optimal strategy.

Another unresolved issue in ERP attention research is whether spatial attention can operate in foveal vision. A number of fMRI studies have demonstrated the ability of attention to modulate activity corresponding to visual field areas both inside and outside foveal vision (e.g. Tootell *et al.*, 1998; Brefczynski & DeYoe, 1999). These studies used multi-element displays with subjects required to attend to some stimuli while ignoring others. In a recent EEG study (Handy & Khoe, 2005), however, attention-related increases in the P1 ERP component were found only to target locations in parafoveal vision (2.2° from fixation), with no similar effect observed to targets located within foveal vision. This study used a cuing paradigm with single stimulus presentations, which may explain the incongruity between the fMRI and EEG findings. While the paradigm described in the current study was not designed to address this question, it is clear that the P1 modulations observed herein and the flexibility of the VESPA method would facilitate the design of a paradigm that would be suitable to investigate this further.

In summary, we have utilized the novel VESPA method to examine the electrophysiological effects of visual spatial attention to multiple, continuously presented stimuli. This has allowed us to resolve the timing of the deployment of endogenous attention in isolation. The strong modulations found in the 90–100-ms range demonstrate that, in the current paradigm, endogenous attention is expressed in modulation of activity during early stages of sensory processing.

## Abbreviations

EEG: electroencephalogram
EOG: electrooculogram
ERP: event-related potential
fMRI: functional magnetic resonance imaging
HEOG: horizontal electrooculogram
SNR: signal-to-noise ratio
SSVEP: steady-state visual-evoked potential
VEP: visual-evoked potential
VESPA: visual-evoked spread spectrum analysis.

## Supplementary material

The following supplementary material may be found on http://www.blackwell-synergy.com

Appendix S1 The blind source separation method.

Video S1 A video illustrating the experiment.
