# Supplementary material for: Isolating endogenous visuo-spatial attentional effects using the novel visual-evoked spread spectrum analysis (VESPA) technique
Source: Eur J Neurosci. 2007 Dec;26(12):3536–42. doi: 10.1111/j.1460-9568.2007.05968.x (PMC2239299; doi:10.1111/j.1460-9568.2007.05968.x)
Supplement: Appendix S1 — The blind source separation method. [file ejn0026-3536_Appendix_S1.doc]

###### **Blind Source Separation**

Blind source separation involves the assumption that the time series from each of the *N* EEG channels, x_i_(t) is an instantaneous mixture of *N* unknown sources s_i_(t), via an unknown mixing matrix **A**.

x(t) = **A**s(t) (1)

By performing a particular source separation algorithm one can estimate an unmixing matrix **W** that approximates **A**^-1^ up to a permutation and scaling of its rows, and the vector of the estimated component values, ŝ(t). We chose to use the second-order blind identification (SOBI) algorithm (Belouchrani et al., 1997) to separate the sources, as, in general, algorithms that use summary statistics, such as SOBI, are preferable to instantaneous algorithms when processing data with poor signal-to-noise such as EEG. The SOBI algorithm works by calculating correlations between pairs of sensors at fixed delays by averaging across the dataset making it robust to noise. In this study, SOBI was carried out using the EEGLAB toolbox (Delorme and Makeig, 2004, http://sccn.ucsd.edu/eeglab/).

We performed SOBI on each 40 s, 64-channel EEG data set corresponding to a trial.

We thus obtained a data set of 40 s of activity from each of 64 underlying sources and an estimate of the mixing matrix **A**. The aim was then to identify a subset of sources, whose activity was related to the waveforms modulating each stimulus pair. This was carried out by solving for the VESPA for each of the two pairs of stimuli for each of these 64 *sources*. Sources related to the waveforms modulating each stimulus pair were identified by checking that the SNR of the VESPA response to the given stimulus was > 1. Unrelated sources were zeroed for each stimulus pair. This led to us having two subsets of relevant sources, one for the UL stimulus, s_UL_(t), and one for the LU, s_LU_(t). Each subset of sources was then mapped back to the original EEG electrode domain by multiplication by the estimating mixing matrix **A**,

x_UL(t)_ = **A** s_UL_(t) x_LU(t)_ = **A** s_LU_(t)

For each stimulus pair, the VESPA at each electrode was then solved for using the corresponding “cleaner” EEG electrode domain data set. The resulting SNR for the VESPA, averaged across all trials, subjects and conditions, was found to be 17.7 dB, compared to 12.9 dB in the case where no source separation was performed.
